# Sirt3 is critical for p53-mediated ferroptosis upon ROS-induced stress

**DOI:** 10.1093/jmcb/mjaa074

**Published:** 2020-12-30

**Authors:** Ying Jin, Wei Gu, Weichang Chen

**Affiliations:** 1 Department of Gastroenterology, The First Affiliated Hospital of Soochow University, Suzhou 215006, China; 2 Institute for Cancer Genetics, and Department of Pathology and Cell Biology, and Herbert Irving Comprehensive Cancer Center, College of Physicians & Surgeons, Columbia University, New York, NY 10032, USA; 3 Department of Gastroenterology, Suzhou Ninth People's Hospital, Suzhou 215200, China

Dear Editor,

p53 acts as a transcription factor to modulate various types of cellular processes to suppress tumor development (Tackmann and Zhang, 2017). The exquisite regulation of p53 functions is of vital importance for cell fate decisions. Although it is well accepted that p53-mediated cell-cycle arrest, senescence, and apoptosis serve as major tumor suppression mechanisms, recent studies suggest that other unconventional activities such as ferroptosis are also critically involved in its tumor suppressor function (Li et al., 2012). We and others found that p53 plays an important role in modulating ferroptotic responses through its metabolic targets (Jiang et al., 2015; Jennis et al., 2016; Wang et al., 2016). Ferroptosis is a regulated form of iron-dependent, non-apoptotic cell death characterized by excessive reactive oxygen species (ROS) generation and accumulation of lipid peroxidates (Stockwell et al., 2020). Lipid peroxides are normally eliminated by glutathione peroxidase 4 (GPX4) and its co-factor glutathione (GSH), which convert lipid hydroperoxides to non-toxic lipid alcohols. Thus, ferroptosis can be artificially induced by pharmacological agents that disrupt this lipid repair system, allowing lethal accumulation of lipid peroxides. Such agents include direct GPX4 enzymatic inhibitors, as well as the small molecule erastin, which suppress glutathione synthesis and indirectly suppress GPX4 activation (Stockwell et al., 2020). Although activation of p53 expression is able to modulate ferroptosis induced by GPX4 inhibition in certain cell types (Jiang et al., 2015), p53-mediated ferroptotic responses are also observed upon ROS-induced stress, apparently through a GPX4-independent manner (Chu et al., 2019). Nevertheless, the molecular factors that modulate p53-dependent ferroptosis upon ROS stress need further elucidation. Sirt3 is a NAD-dependent deacetylase predominantly localized in mitochondria, which functions in multiple metabolic pathways, including electron transport chain, fatty acid oxidation, amino acid metabolism, redox balance, and the tricarboxylic acid (TCA) cycle (Van de Ven et al., 2017). Here, we identified Sirt3 as a novel repressor in p53-mediated ferroptosis induced by ROS stress. 

To investigate the role of Sirt3 in modulating p53-mediated ferroptosis, we first examined whether Sirt3 overexpression affects ROS-induced ferroptosis in human osteosarcoma cell line U2OS. To this end, U2OS cells were transfected with a Flag-tagged Sirt3 expression vector (Supplementary Figure S1A). Our previous studies established a p53-dependent ferroptosis response upon ROS-induced stress, in the absence of GPX4 inhibitors such as erastin. As expected, p53-mediated ferroptosis was readily induced in the control U2OS cells upon the treatment of both Nutlin (for p53 activation) and TBH (for increasing ROS levels) (Figure 1A and B, lane 4), suggesting that ferroptotic cell death requires both p53 activation and ROS stress. However, the levels of ferroptosis were significantly reduced by Sirt3 expression (Figure 1A and B, lane 9). As expected, the cell death induced under these conditions were specifically blocked by ferr-1, a well-known ferroptosis inhibitor (Figure 1A and B). Similar results were also obtained in human melanoma cell line A375 (Figure 1C;Supplementary Figure S1B) and human gastric cell line AGS, both of which express wild-type p53 (Supplementary Figure S1C and D), suggesting that Sirt3 expression is able to suppress p53-mediated ferroptosis. To examine the role of endogenous Sirt3 in p53-mediated ferroptosis, we used CRISPR/Cas9 technology to inactivate the Sirt3 gene in U2OS (Supplementary Figure S2A). As shown in Figure 1D, p53-mediated ferroptosis was significantly elevated upon loss of Sirt3 expression. Likewise, p53-mediated ferroptosis was also markedly enhanced by Sirt3 depletion in AGS cells (Supplementary Figure S2B and C). These data indicate that inactivation of Sirt3 significantly sensitizes cells to p53-dependent ferroptosis upon ROS-induced stress.

**Figure 1:**
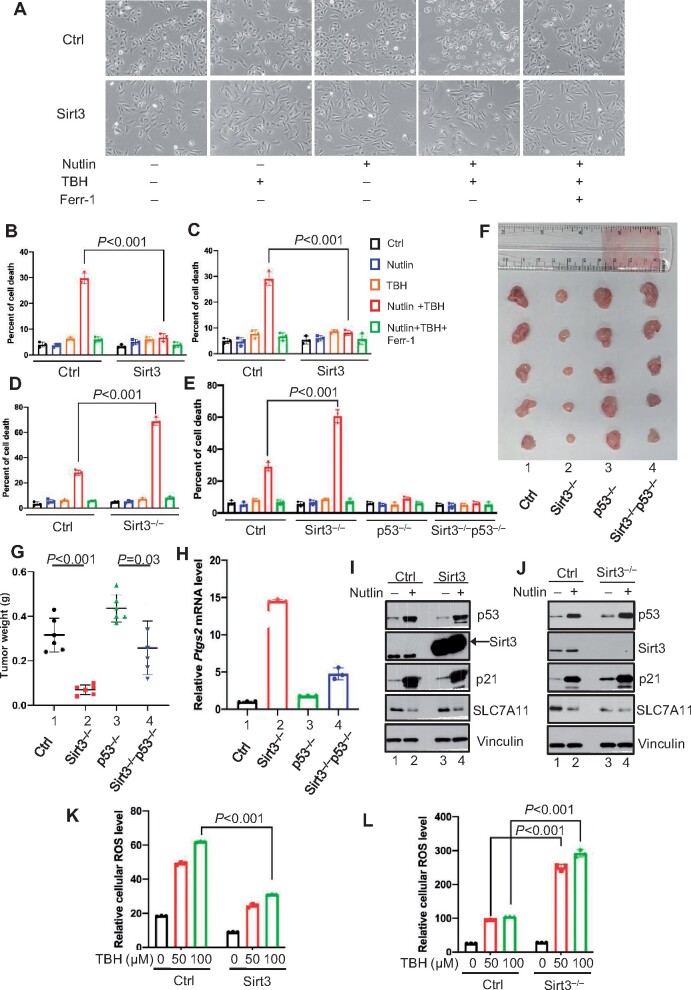
The roles of Sirt3 in modulating p53-mediated ferroptosis and tumor suppression. (**A**) Representative phase-contrast images of control and Sirt3-overexpressed U2OS cells in ferroptosis analysis. Cells were treated with TBH, Nutlin, or Ferr-1 as indicated. The experiments were repeated twice, independently, with similar results. (**B**–**E**) Ferroptosis assays for control and Sirt3-overexpressed U2OS cells (**B**), control and Sirt3-overexpressed A375 cells (**C**), control and Sirt3^–/–^ U2OS cells (**D**), as well as control, Sirt3^–/–^, p53^–/–^, and Sirt3^–/–^p53^–/–^ A375 cells (**E**). Cells were treated with TBH, Nutlin, or Ferr-1 as indicated. Mean ± SD is shown; *n *=* *3 independent experiments. (**F**–**H**) Mouse xenograft tumor models were established by injection of control, Sirt3^–/–^, p53^–/–^, and Sirt3^–/–^p53^–/–^ A375 cells. (**F**) Xenograft tumors were harvested after three weeks as indicated. (**G**) Tumor weights were determined (error bars, from five tumors). (**H**) qPCR of *Ptgs2* mRNA levels in harvested tumors. (**I** and **J**) Western blotting analysis of the cell extracts from control and Sirt3-overexpressed U2OS cells (**I**) or control and Sirt3^–/–^ cells (**J**) treated with Nutlin or not, by antibodies indicated. The experiments were repeated twice, independently, with similar results. (**K** and **L**) Control and Sirt3-overexpressed U2OS cells (**K**) or control and Sirt3^–/–^ A375 cells (**L**) were treated with either DMSO (vehicle) or TBH for 1 h, and then stained with H2DCFDA for cellular ROS assays.

To further validate the role of Sirt3 in p53-mediated ferroptosis, we used the CRISPR/Cas9 method to inactivate the *Sirt3* gene in human melanoma A375 cells and isogenic p53-null A375 cells (Supplementary Figure S2D). Consistent with above data, loss of Sirt3 significantly sensitized the ferroptotic responses in A375 cells; however, loss of Sirt3 expression had no obvious effects on ferroptosis under the same conditions in p53-null A375 cells (Figure 1E), indicating that Sirt3 indeed critically regulates p53-mediated ferroptosis in human cancer cells. Next, to examine the potential oncogenic role of Sirt3 *in vivo*, we tested whether loss of Sirt3 expression affects p53-mediated tumor growth suppression by using xenograft tumor models. Indeed, the tumor growth of human melanoma A375 cells was dramatically reduced upon inactivation of Sirt3 expression (Figure 1F and G, lane 2 vs. lane 1) in xenograft tumor growth assays; nevertheless, the tumor-suppressive effects induced by loss of Sirt3 expression were largely abrogated when isogenic p53-null A375 cells were used in xenograft tumor growth (Figure 1F and G, lane 4 vs. lane 3). Notably, upregulation of *Ptgs2*, a marker of ferroptosis, was observed in Sirt3^–/–^ tumor samples but largely abrogated in Sirt3^–/–^p53^–/–^ samples (Figure 1H). Taken together, these data demonstrate that inactivation of Sirt3 significantly enhances p53-mediated ferroptosis upon ROS stress and this upregulation, at least, in part contributes to p53-dependent tumor growth suppression.

It is well accepted that Sirt1 plays an important role in regulating p53-mediated transcriptional activation by deacetylating p53 (Zhao et al., 2008). Unlike Sirt1, Sirt3, predominately localized in mitochondria, apparently does not directly interact with p53 (Zhao et al., 2008). Consistent with this notion, neither Sirt3 overexpression nor loss of endogenous Sirt3 expression had any significant effect on the levels of p53 as well as its activation of p21 (Figure 1I and J). Likewise, p53-mediated repression of SLC7A11 also remained intact (Figure 1I and J). Ferroptosis is triggered by accumulation of lipid peroxidation, which is tightly regulated by ROS levels in the cell (Chu et al., 2019). Since Sirt3 is critically involved in regulating ROS responses through several metabolic enzymes (Zhang et al., 2020), we examined the role of Sirt3 in modulating the cellular ROS levels. As shown in Figure 1K, Sirt3 overexpression significantly reduced the ROS levels in cells under different concentrations of TBH treatment; conversely, the ROS levels were increased dramatically upon loss of Sirt3 expression (Figure 1L). Taken together, these data suggest that SirT3 regulates p53-dependent ferroptosis by modulating cellular ROS levels without affecting p53 levels and p53-mediated transcriptional activity.

Among multiple layers of mechanisms controlling p53 function, negative regulators in modulating p53 responses such as Mdm2/Mdmx oncoproteins are particularly interesting, as these factors act as potential targets for activating p53 function in cancer therapy (Tackmann and Zhang, 2017). Our data shown above identified Sirt3 as a critical repressor of ROS-induced cell death responses mediated by p53. We found that overexpression of Sirt3 effectively suppresses p53-dependent ferroptosis upon ROS stress. In contrast, depletion of endogenous Sirt3 sensitizes the cells for ferroptotic cell death. Notably, Sirt3 does not affect p53-mediated transcriptional activity but, instead, regulates p53-dependent ferroptosis through modulating cellular ROS levels in human cancer cells. Finally, by using xenograft mouse models, we further showed that inactivation of Sirt3 significantly enhances p53-mediated tumor growth suppression *in vivo*. Taken together, our study demonstrates that Sirt3 acts a novel regulator in p53-mediated ferroptosis and tumor suppression.

The molecular mechanism by which inactivation of Sirt3 promotes p53-mediated tumor growth suppression needs further elucidation. As expected, western blotting analysis revealed that high levels of SLC7A11 were expressed in p53-null A375 whereas the levels of SLC7A11 were downregulated in control A375 cells expressing wild-type p53 (Supplementary Figure S3A). Interestingly, although loss of Sirt3 modestly increased the levels of ROS in p53^–/–^Sirt3^–/–^ A375 cells, much higher levels of ROS were detected in wild-type p53-expressing A375 cells upon loss of Sirt3 (Supplementary Figure S3B). These data indicate that loss of Sirt3 expression makes p53-expressing cells more vulnerable to the ROS stress. Since SLC7A11 is required for cystine update, which is critical for the synthesis of GSH to detoxify ROS-induced damage, p53-mediated downregulation of SLC7A11 abrogates the ability of cancer cells to neutralize the effects induced by the ROS stress. On the other hand, Sirt3 is the major deacetylase within the mitochondrial matrix and activates several mitochondrial proteins including isocitrate dehydrogenase 2 (IDH2) and superoxide dismutase 2 (SOD2, alias MnSOD) through deacetylation (Van de Ven et al., 2017). For example, IDH2 catalyzes the TCA cycle redox conversion of isocitrate to α-ketoglutarate and serves as a major source of NADPH production, which is critical for the ratio of GSH to GSSG. SOD2 is directly involved in neutralizing ROS levels (Zhang et al., 2020). Since Sirt3-mediated deacetylation is critical for the activities of IDH2 or SOD, inhibition of Sirt3 leads to suppression of IDH2-mediated NADPH production and SOD2-dependent neutralization of ROS levels. Thus, the combination of p53-mediated downregulation of SLC7A11 and inactivation of Sirt3 makes the cells more sensitive to ROS-induced ferroptosis for tumor growth suppression. Accumulating evidence reveals that ferroptosis acts as a novel mechanism in tumor suppression independent of classic tumor-suppressive activities (Stockwell et al., 2020). Notably, p53-mediated ferroptoic responses were significantly enhanced upon the treatment of LC-0296 (Alhazzazi et al., 2016), a specific inhibitor of Sirt3 (Supplementary Figure S4A and B). Although further investigations are clearly required, given that inactivation of Sirt3 significantly promotes the tumor growth suppression of p53, Sirt3 is an attractive target for cancer therapy at least in certain types of human cancers.

*[Supplementary material is available at Journal of Molecular Cell Biology online. We thank Huan Li for help in establishing cell lines and Xin Yang and Yanqing Liu for comments on the manuscript. Conception and experimental design: Y.J., W.C., and W.G. Methodology and data acquisition: Y.J., W.C., and W.G. Analysis and interpretation of data: Y.J., W.G., and W.C. Manuscript writing: Y.J., W.G., and W.C.]*

## Supplementary Material

mjaa074_Supplementary_Data
