# Supplementary material for: Sirt3 is critical for p53-mediated ferroptosis upon ROS-induced stress
Source: J Mol Cell Biol. 2020 Dec 30;13(2):151–4. doi: 10.1093/jmcb/mjaa074 (PMC8104950; doi:10.1093/jmcb/mjaa074)
Supplement: mjaa074_Supplementary_Data [file mjaa074_supplementary_data.pdf]

Supplementary material

Sirt3 is critical for p53-mediated ferroptosis upon ROS-induced stress

Ying Jin[1,2,3], Wei Gu[2,*], and Weichang Chen[1,*]

[1] Department of Gastroenterology, The First Affiliated Hospital of Soochow University, Suzhou 215006, China

[2] Institute for Cancer Genetics, and Department of Pathology and Cell Biology, and Herbert Irving Comprehensive Cancer Center, College of Physicians & Surgeons, Columbia University, New York, NY 10032, USA

[3] Department of Gastroenterology, Suzhou Ninth People's Hospital, Suzhou 215200, China

* Correspondence to: Weichang Chen, E-mail: weichangchen@126.com; Wei Gu, E-mail: wg8@cumc.columbia.edu; Tel: +1-212-851-5282; Fax: +1-212-851-5284

Materials and methods

Cell culture and stable line generation

The U2OS, A375, and AGS cancer cell lines were obtained from the American Type Culture Collection (ATCC) and have been proven to be negative for mycoplasma contamination. The cell lines used in this work were not listed in the ICLAC database. All cells were cultured in a 37°C incubator with 5% CO2. All media used were supplemented with 10% FBS, 100 units/ml penicillin and 100 mg/ml streptomycin. U2OS and A375 cells were maintained in DMEM medium (Gibco) and AGS cells were maintained in F-12K medium (from ATCC). To obtain SIRT3 knockout cell lines, the cells were transfected with a SIRT3 double nickase plasmid (sc-400675-NIC; Santa Cruz) using Lipofectamine 3000 (Invitrogen), and then were selected with puromycin (U2OS and A375 with1 µg/ml, AGS with 0.5 µg/ml) for 3–6 days. SIRT3 knockout single clones were screened by western blotting after continuing to culture 2–3 weeks without selective antibiotics. To generate Sirt3$^{-/-}$p53$^{-/-}$ double knockout cells, the same strategies were used (p53, sc-416469-NIC). To obtain the Sirt3 stable cell lines, pcDNA3.1-SIRT3-Flag was transfected into U2OS, A375, and AGS cells for selection and maintenance with 1 mg/ml G418 (Sigma). Single clones were selected and screened by western blotting.

Western blotting and antibodies

Proteins were lysed from cancer cells by using Flag lysis buffer [50 mM Tris-Cl (pH=7.3), 137 mM NaCl, 10mM NaF, 1 mM NaVO4, 10% Glycerol, 0.5 mM EDTA, 1% Triton X-100, 0.2% Sarkosyl] with fresh protease inhibitor (1:1000). Protein extracts were resolved on 4%–20% bis-tris gels (Invitrogen), then transferred onto nitrocellulose membranes, blocked with 5% skim milk in TBST for 0.5 h, and incubated with primary antibodies at 4°C overnight. Membranes were incubated with HRP-conjugated secondary antibodies for 1 h at room temperature after washing. The western blot signals were detected on autoradiographic films after incubating with ECL (Thermo scientific) or Super Signal West Dura reagents (Thermo scientific). Primary antibodies recognized p53 (sc-126; Santa Cruz), p21 (sc-53870; Santa Cruz), SLC7A11 (12691s; Cell Signaling Technology), SIRT3 (267s; Cell Signaling Technology), and vinculin (V9264; Sigma-Aldrich).

RNA extraction and quantitative RT–PCR

Total RNA was isolated by using TRIzol (Invitrogen) according to the manufacturer's

protocol. cDNA was reverse-transcribed by using SuperScript IV First-Strand Synthesis (Invitrogen) also according to the manufacturer's protocol. For PCR analysis, the Applied Biosystems 7500 Fast System was used. For the qRT-PCR analysis, the following primers were used: human PTGS2 forward 5′-CTTCACGCATCAGTTTTTCAAG-3′, PTGS2 reverse 5′-TCACCGTAAATATGATTTAAGTCCAC-3′; human GAPDH forward 5′-ATCAATGGAAATCCCATCACCA-3′, GAPDH reverse 5′-GACTCCACGACGTACTCAGCG-3′.

Drugs and inhibitors

For ROS generation, TBH (Sigma) was used at different doses. Nutlin (Sigma) was used in experiments at a concentration of 10 μM. Ferrostatin-1 (ferroptosis inhibitor; Xcess Biosciences), 4 μM; N-acetyl-cysteine (NAC; Sigma) 10 mM; LC-0296 (Sirt3 inhibitor; AOBIOUS) at different doses depending on the experiment.

Ferroptosis assays

The ferroptosis assays were basically performed as described previously (Jiang et al., 2015; Chu et al., 2019). p53-dependent ferroptosis requires both p53 activation and ROS stress. Cells were treated with Nutlin (10 μM) for 24 h first for p53 pre-activation then followed by treatment with TBH (U2OS 400 μM, A375 250 μM, AGS 200 μM) for 8 h. For quantification of cell death, cells were trypsinized, collected, and stained with trypan blue followed by using a cell number counter (Life Technologies countess II) according to the manufacturer's protocol. The cells were also treated with Ferr-1 to confirm whether the cell death is ferroptotic cell death.

Analysis of ROS production

Cells were incubated with PBS containing 10 μM H2DCFDA (from Invitrogen) at 37°C for 30 min in the incubator. Then cells were washed, incubated with DMEM containing TBH (at different doses), and returned to the incubator for 30 min again. Cells were harvested and washed with PBS followed by resuspending in 500 μl PBS. ROS levels were analyzed using a Becton Dickinson FACS Calibur machine through the FL1 channel, and the data were analyzed using FlowJo. For each sample, 10000 cells were analyzed.

Mouse xenograft

Control, $Sirt3^{-/-}$, $p53^{-/-}$, and $Sirt3^{-/-}p53^{-/-}$ A375 cells were trypsinized and counted. Then,

1.0×10^6 cells were mixed with Matrigel (BD Biosciences) at 1:1 (vol:vol) ratio and injected subcutaneously into nude mice (NU/ NU; Charles River). Three weeks after injection, mice were sacrificed, and tumors under the skin were dissected.

Quantification and statistical analysis

Results were shown as mean ± SD. Microsoft Excel software and GraphPad Prism were used for statistical analysis. Statistical significance was determined by using a two-tailed, unpaired Student's *t*-test with a confidence interval of 95%. $P<0.05$ was denoted as statistically significant.

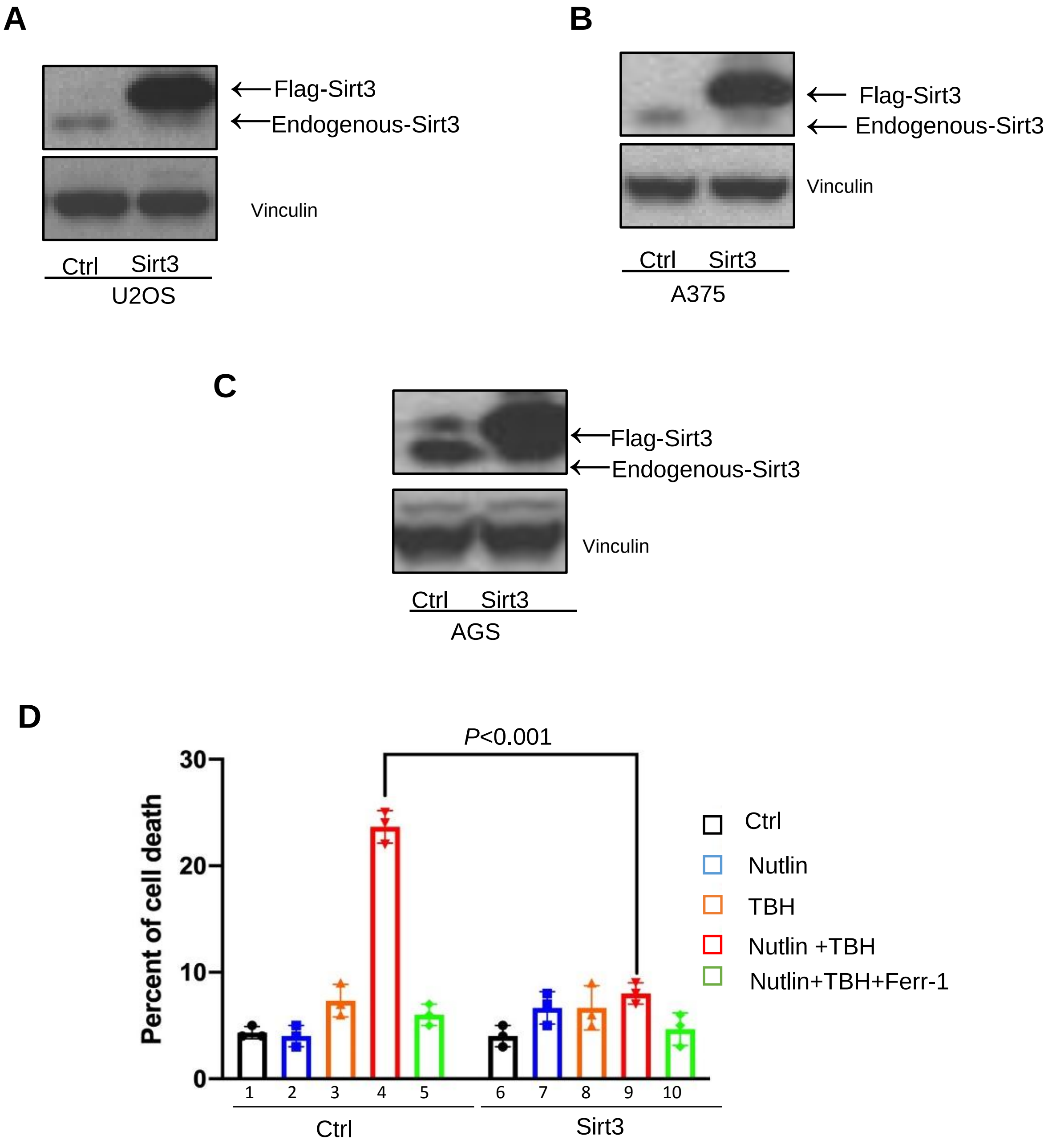

Supplementary Figure S1 Effects of Sirt3 overexpression on ferroptosis. (**A**) Western blotting analysis of the cell extracts from control and Sirt3-overexpressed U2OS cells, by the antibodies as indicated. (**B**) Western blotting analysis of the cell extracts from control or Sirt3-overexpressed A375 cells, by the antibodies as indicated. (**C**) Western blotting analysis of the cell extracts from control or Sirt3-overexpressed AGS cells, by the antibodies as indicated. (**D**) Ferroptosis assays for control and Sirt3-overexpressed AGS cells. The cells were treated with TBH, Nutlin, or Ferr-1 as indicated. Mean± SD is shown; *n*=3 independent experiments.

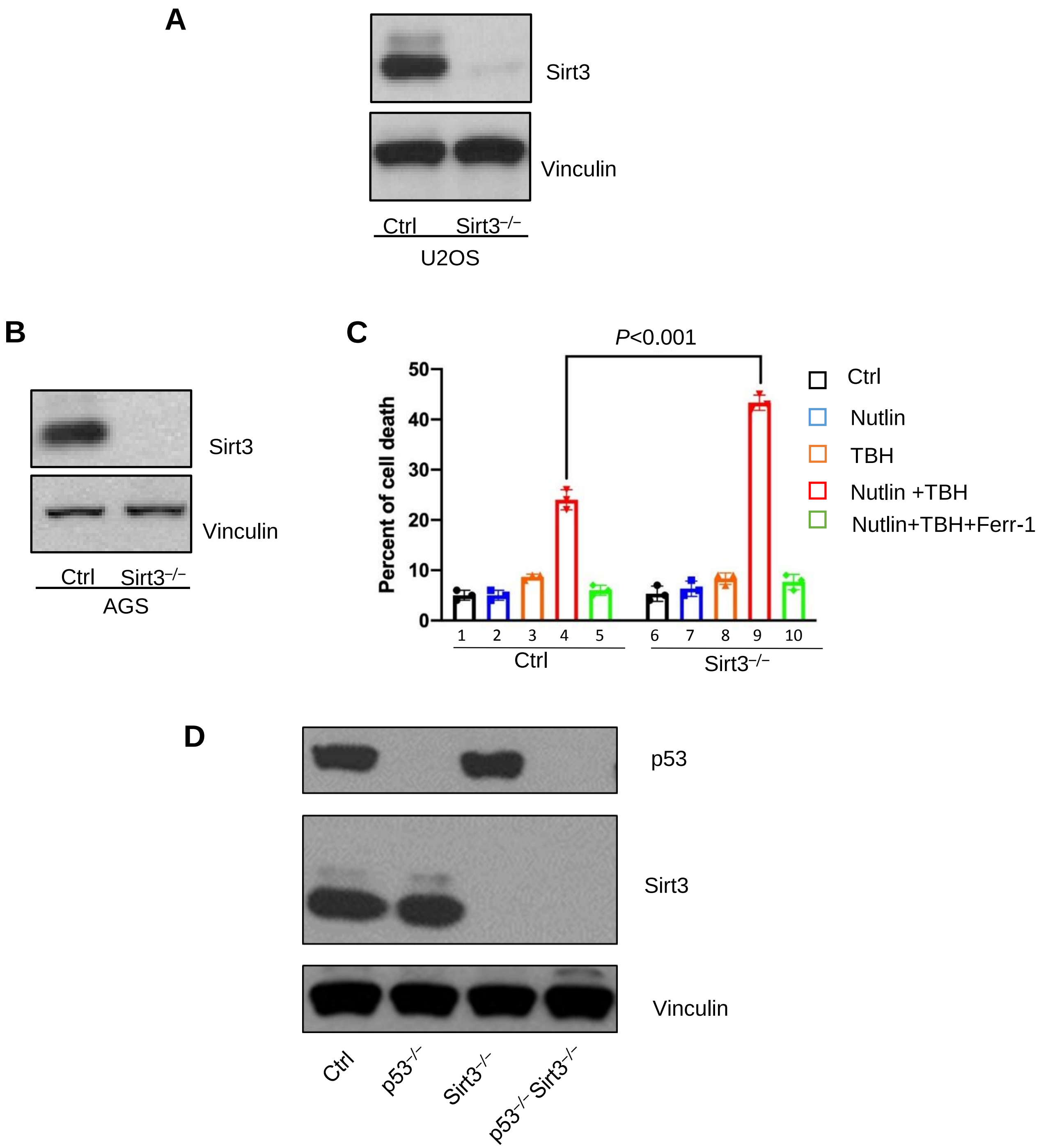

Supplementary Figure S2 Loss of Sirt3 expression sensitizes p53-mediated ferroptosis. (**A**) Western blotting analysis of the cell extracts from control and Sirt3$^{-/-}$ U2OS cells, by the antibodies as indicated. (**B**) Western blotting analysis of the cell extracts from control and Sirt3$^{-/-}$ AGS cells, by the antibodies as indicated. (**C**) Ferroptosis assays for control and Sirt3$^{-/-}$ AGS cells. The cells were treated with TBH, Nutlin, or Ferr-1 as indicated. Mean± SD is shown; *n*=3 independent experiments. (**D**) Western blotting analysis of the cell extracts from control or knockout A375 cells, by the antibodies as indicated.

A

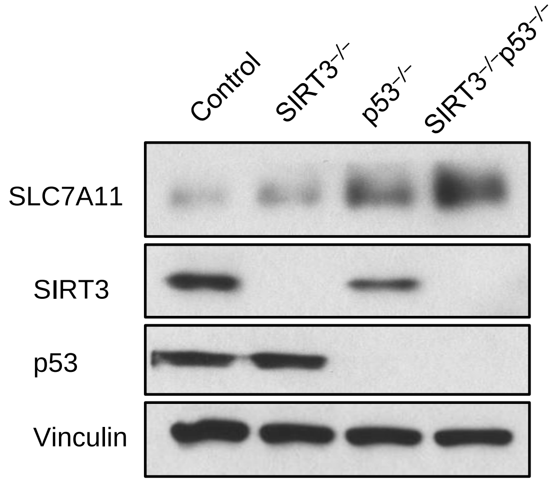

B

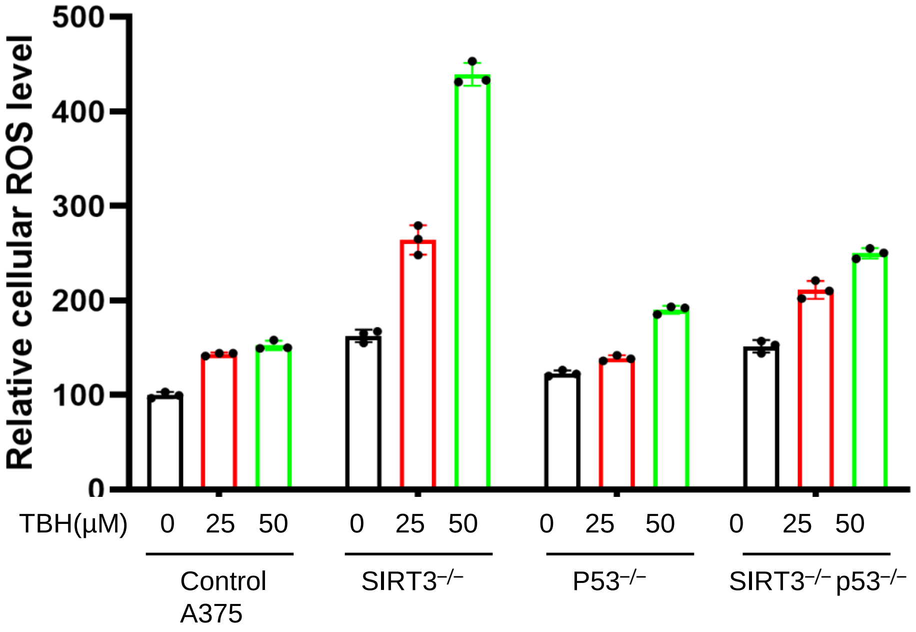

Supplementary Figure S3 Effects of Sirt3 and p53 on ROS levels. (**A**) Western blotting analysis of the cell extracts from control A375, Sirt3$^{-/-}$ A375, p53$^{-/-}$A375, and p53$^{-/-}$Sirt3$^{-/-}$ A375 cells, by the antibodies as indicated. (**B**) The levels of ROS in control A375, Sirt3$^{-/-}$ A375, p53$^{-/-}$ A375, and p53$^{-/-}$Sirt3$^{-/-}$ A375 cells upon the treatment of TBH as indicated.

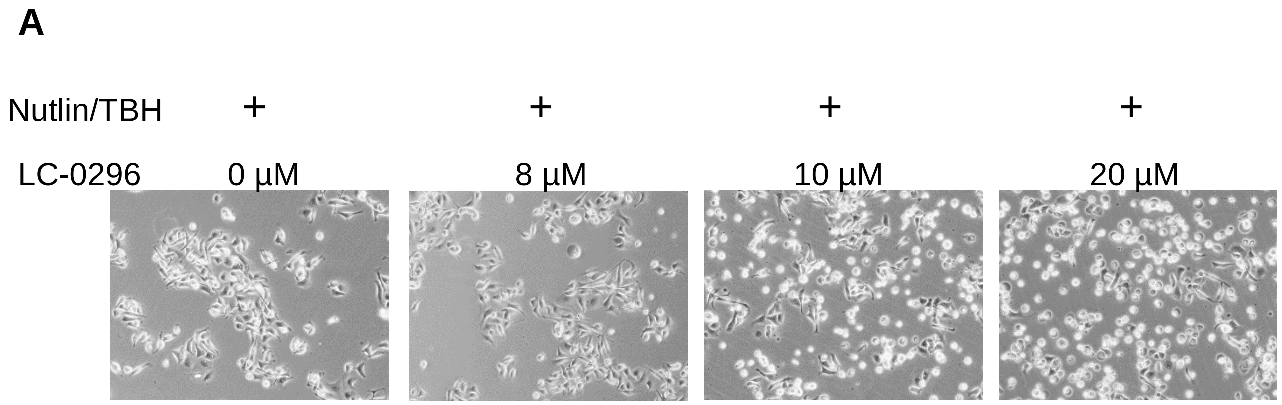

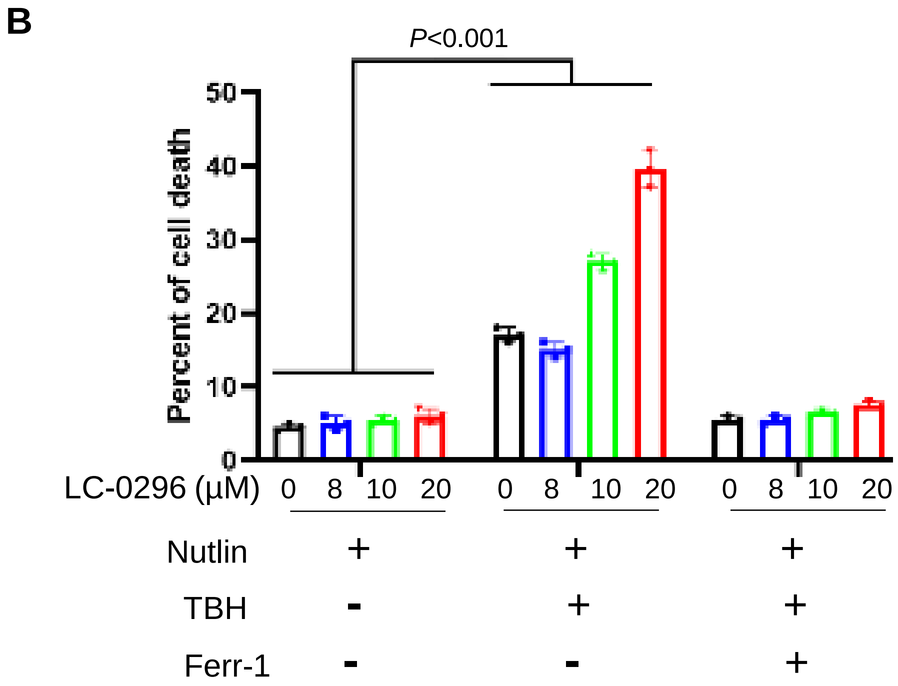

Supplementary Figure S4 Effects of a Sirt3 inhibitor on ferroptosis. (**A**) Representative phase-contrast images of AGS cells in ferroptosis analysis. Cells were treated with TBH, Nutlin, and either DMSO (control) or LC-0296 as indicated. The experiments were repeated twice, independently, with similar results. (**B**) AGS cells were treated with TBH, Nutlin, LC-0296, or Ferr-1 as indicated. Mean± SD is shown; *n*=3 independent experiments.
